# Obesity and its associations with autonomic and cognitive functions in the general population

**DOI:** 10.1371/journal.pone.0322802

**Published:** 2025-05-08

**Authors:** Battuvshin Lkhagvasuren, Zhiping P. Pang, Tsolmon Jadamba, Tetsuya Hiramoto, Keely Cheslack–Postava, George J. Musa, Christina W. Hoven, Nobuyuki Sudo

**Affiliations:** 1 Brain and Mind Research Institute, Mongolian Academy of Sciences, Ulaanbaatar, Mongolia; 2 Brain Science Institute, Mongolian National University of Medical Sciences, Ulaanbaatar, Mongolia; 3 Department of Psychosomatic Medicine, Graduate School of Medical Sciences, Kyushu University, Fukuoka, Japan; 4 Center for NeuroMetabolism, Child Health Institute of New Jersey, Department of Neuroscience and Cell Biology, Robert Wood Johnson Medical School, Rutgers University, New Brunswick, New Jersey, United States of America; 5 Department of Psychosomatic Medicine, NHO Fukuoka National Hospital, Fukuoka, Japan; 6 Global Psychiatric Epidemiology Group, Division of Child and Adolescent Psychiatry, Department of Psychiatry, Columbia University-New York State Psychiatric Institute, New York, United States of America; 7 Department of Epidemiology, Columbia University, Mailman School of Public Health, New York, New York, United States of America; 8 Department of Child and Adolescent Psychiatry, New York State Psychiatric Institute, New York, New York, United States of America; University of Bucharest, Faculty of Biology, ROMANIA

## Abstract

**Background:**

Obesity poses a significant global health burden. This study aimed to investigate the prevalence of obesity in Mongolia and its associations with autonomic and cognitive functions while considering potential psychosocial risk factors.

**Methods:**

This population-based, cross-sectional study included 382 participants who underwent physical examinations, completed health-related questionnaires, and participated in heart rate variability (HRV) testing for autonomic assessment and the mini-mental state examination for cognitive evaluation.

**Results:**

Obesity prevalence was 28.1% (age-sex adjusted). Individuals with obesity were more likely to be older, married, have lower education, and engage in less physical activity. They exhibited autonomic imbalance, decreased autonomic nervous system activity, lower cognitive function, and sleep disturbances compared to the individuals without obesity. Body mass index, and waist circumference inversely correlated with HRV indices. Female sex, lower education, apartment living, alcohol consumption, sleep disturbances, and autonomic dysfunction emerged as significant risk factors for obesity. Independent predictors of autonomic dysfunction included systolic blood pressure, physical activity, and neck circumference, while age, education, height, sleep apnea, and autonomic dysfunction predicted cognitive decline. Furthermore, generalized linear mediation models revealed a partial mediation effect of autonomic dysfunction on the association between obesity and cognitive decline.

**Conclusion:**

This study highlights a high prevalence of obesity in the general population (28.1%) and identifies distinct characteristics associated with the condition. Furthermore, our findings suggest a potential indirect effect of obesity on cognitive function, mediated by autonomic dysfunction. Further research is needed to elucidate the causal relationships and develop targeted interventions for high-risk groups (females, individuals with lower education) and promotion initiatives of healthy lifestyles (less alcohol, exercise, and sleep hygiene) to address both obesity and its associated health complications, including autonomic dysfunction.

## 1 Introduction

Obesity has become a critical public health concern for children, adolescents, and adults worldwide [1]. Since 1975, the global prevalence of obesity has more than tripled, with nearly 2.5 billion adults classified as overweight and over 890 million considered obese [2]. Furthermore, this issue affects approximately one in five children and adolescents [3]. According to the Global Burden of Disease study, 4.7 million premature deaths in 2017 were attributed to obesity [4]. The prevalence of central obesity, defined as excess adipose tissue in the abdominal region, has increased globally, particularly among young and male individuals [5]. In Mongolia, the prevalence of central obesity in the general population was 22.5% in 2023, and the prevalence of obesity was 26.4% in 2013 [6,7]. The 2019 STEPS survey found higher rates of central obesity, and the prevalence of overweight and obesity among adults was 36.8% and 14.7%, respectively [8].

It’s well known that obesity can significantly impact an individual’s quality of life and psychological well-being [9]. Our previous study demonstrated that individuals with obesity reported lower quality of life compared to those without obesity [10]. Obesity is associated with several comorbidities, including metabolic syndrome, cardiovascular disease, autonomic dysfunction, and cognitive decline [11–15].

Furthermore, the autonomic nervous system plays a crucial role in regulating the body’s physiological functions. In individuals with obesity, autonomic dysfunction has been observed, characterized by increased sympathetic activity and decreased parasympathetic activity [13,16]. Furthermore, evidence indicated that autonomic dysfunction in obesity could result in reduced thermogenesis, decreased energy expenditure, and impaired glucose uptake in skeletal muscle [17]. Studies have also demonstrated autonomic dysfunction in obesity can lead to altered intestinal motility, which contributes to gut dysbiosis and increased inflammation [13,18]. Conversely, chronic gastrointestinal inflammation has been demonstrated to alter the composition of the gut microbiota, which may contribute to the development of autonomic dysfunction [19,20]. This complex relationship between obesity, inflammation, and gut health is becoming increasingly recognized [21–23]. Possible mechanisms underlying this detrimental relationship might be hypothalamic inflammation in the brain, in which the specific nuclei for eating behaviors are located. Pro-inflammatory signaling, including diet-derived saturated fatty acids and prostaglandins, through the median eminence activates the arcuate nucleus, a satiety control center, which may lead to develop obesity [14]. Additionally, obesity is often accompanied by sarcopenia, a condition characterized by muscle loss. This combination can exacerbate metabolic dysfunction and contribute to poorer cognitive and physical outcomes [24–26].

Despite the link between obesity and cognitive decline is well-established, the precise mechanisms underlying this association remain unclear [27]. The autonomic dysfunction has been proposed as a potential mechanism contributing to cognitive decline in obesity by affecting cerebral blood flow, neurotransmitter signaling, and the neuroinflammation [14]. Some studies have suggested an association between heart rate variability (HRV) and impaired cognitive function in individuals with obesity [28,29]. HRV is reliable, a non-invasive measure of autonomic function that has been investigated as a potential biomarker for autonomic dysfunction [30]. HRV data can be obtained by recording the beat-to-beat heart rate using Holter electrocardiography for long-term evaluation. Alternatively, portable HRV devices are frequently used for screening purposes. Beat-to-beat heart rate variations provide insights into the modulation of the cardiac autonomic nervous system [31]. Irregular HRV findings are associated with increased mortality, hypertension, and type II diabetes [16].

However, there is limited evidence from population-based studies on how obesity affects autonomic and cognitive function. Methodological challenges, including the requirement for specialized equipment to assess autonomic function, and the confounding effects of comorbidities and obesity phenotypes, may contribute to the scarcity of population-based studies on the relationship between obesity, autonomic dysfunction, and cognitive function.

In this study, we aimed to determine the current prevalence of obesity and its associations with autonomic and cognitive function in the general population, while accounting for potential risk factors.

## 2 Materials and methods

### 2.1 Study participants and recruitment

**Fig 1:**
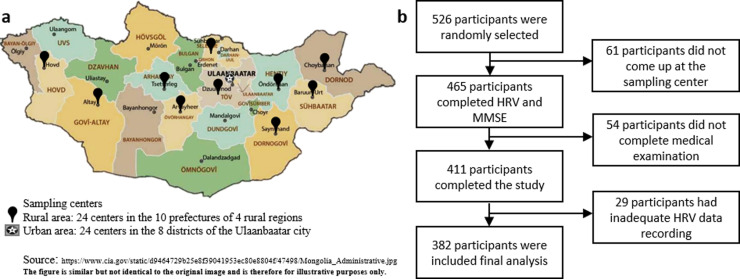
Study flowchart. **a)** Sampling sites across the country. The study consisted of 48 sampling centers, including 24 centers in Ulaanbaatar and 24 in 4 rural regions. **b)** Study flowchart: The required sample size was 385. We invited 540 individuals to participate in this study. Among them, 75 did not show up, 54 participants were excluded due to the missing data and 29 participants were inadequate HRV data recordings. A total of 382 participants were included in the final analysis.

This cross-sectional study was conducted as part of a nationwide multicenter, interdisciplinary, prospective, population-based cohort study in the general population of Mongolia [32]. The sampling size was calculated to be 385 using a confidence level of 95%, margin of error of 5%, design effect of 1.50, and population size of 1,910,630 individuals aged between 18 and 65 years in Mongolia [33]. Expecting a response rate of 60%, a total of 540 individuals who fulfilled the inclusion criteria were invited to participate in the study. The criteria included anyone: *i)* who was a Mongolian citizen; *ii)* who lived in a geopolitical unit for at least 6 months; *iii)* who did not receive health care services during the study. A multi-stage sampling technique was employed to ensure a representative sample. First, eleven geopolitical units were selected: Ulaanbaatar and ten rural prefectures (Gobi-Altai, Khovd, Uvurkhangai, Arkhangai, Selenge, Tuv, Dornogobi, Khentii, Dornod, and Sukhbaatar), as shown in Fig 1a. Second, 48 sampling centers were identified within these units, with 24 in Ulaanbaatar and 24 in rural areas. Sampling centers were selected based on population size, geographic distribution, and accessibility. Finally, individuals meeting the inclusion criteria were randomly selected from each center. Among the invited individuals, 75 did not present themselves at the sampling center on the designated date and time. Of the 465 participants who completed the HRV test and Mini-Mental State Examination (MMSE), 54 had missing data on the medical examination and 29 had inadequate HRV data recordings. The remaining 382 participants were included in the final analysis (Fig 1b).

### 2.2 Data collection and procedures

The data collection started on August 3 2020, and was completed on November 29, 2020. Trained research personnel and medical doctors conducted the study with participants. They explained the procedures to the participants face-to-face. Sociodemographic and lifestyle characteristics, which included 9 sociodemographic and 6 lifestyle determinants, were collected using a tablet. Furthermore, health-related quality of life and psychological conditions were evaluated with self-report questionnaires using a tablet. After completing self-report questionnaires, participants underwent physical examination of their body composition and vital function signs. Autonomic function was assessed using HRV testing and cognitive function was evaluated using the MMSE. The study was conducted in the official language (Mongolian). Informed consent was obtained from the total participants after the objectives, procedure, and instruments of the study had been explained. To protect participant confidentiality, data were collected using identifiers known only to the research team. Once data collection was complete, identifiers were removed, and data were coded to ensure participant anonymity. All information related to study participants will remain confidential.

### 2.3 Measurements

#### Sociodemographic and lifestyle assessment.

Sociodemographic data (age, sex, marital status, education level, employment status, income, workplace safety, living condition, and residential location) and lifestyle determinants (alcohol consumption, smoking status, physical activity level, vegetable consumption, fruit consumption, and food allergy) were collected using a standardized questionnaire administered electronically on a tablet computer.

#### Health-related quality *of* life.

The health-related quality of life was assessed using the World Health Organization Quality of Life-Brief (WHOQOL-BREF), a 24-item questionnaire that has been translated into more than 40 languages. The WHOQOL-BREF determines four domains of the quality of life: physical, psychological, social relationships, and environmental [34]. The Mongolian version of the WHOQOL-BREF demonstrated good psychometric properties in our previous study [10,35].

#### Psychological conditions.

We assessed psychological conditions using self-report questionnaires for psychological symptoms and sleep disturbance. Psychological symptoms were assessed using The Hospital Anxiety and Depression Scale (HADS). The 14-item self-report questionnaire assesses anxiety and depression symptoms, which have been demonstrated to significantly impact metabolic processes and autonomic nervous system function [36,37]. It has seven items for anxiety (HADS-A) and seven items for depression (HADS-D) [38]. The HADS has been translated into Mongolian and has good psychometric properties [39].

#### Sleep dysfunction.

Sleep dysfunction was assessed using the Pittsburgh Sleep Quality Index (PSQI) and the Berlin Questionnaire (BQ). The PSQI is a 19-item instrument that measures seven domains of sleep quality [40]. The PSQI has been widely used as a valid scale for sleep quality studies in clinical and non-clinical populations [40,41]. In our previous study, we adapted the tool in the Mongolian general population [42]. The BQ is a 10-item questionnaire that assesses risk for obstructive sleep apnea [43]. The BQ has been extensively studied and validated in clinical and general populations, and has good sensitivity for the screening of obstructive sleep apnea [44].

#### Body measures and vital signs.

All procedures were non-invasive and performed by trained research personnel or medical doctors. To determine the current physical health status, we examined four primary vital function signs, including body temperature (an electronic infrared thermometer, forehead skin, Tida TD-133, China), blood pressure, and heart rate (an advanced blood pressure monitor, arm, Microlife BP A6 PC, Switzerland). Body height, weight, waist circumference, and neck circumference were measured and the BMI, the ratio of body weight to squared, was calculated. Obesity was defined by a BMI of 30 kg/ m^2^ or greater [45], and central obesity was defined as a waist circumference of greater than 94 cm or greater for men and 80 cm or greater for women, according to the WHO guidelines [46].

#### Autonomic function assessment.

Autonomic function assessment was evaluated using the HRV examination. HRV is a non-invasive measure of the variation in time between successive heartbeats, and it provides important information about the balance and interplay between the sympathetic and parasympathetic branches of the autonomic nervous system [47]. We used a handheld HRV device (CMH V3.0, DailyCare Biomedical Inc., Taoyuan, Taiwan) that records the electrocardiographic data from each participant in a short-term resting phase for a 5-min HRV assessment and extracts the data by the standard time-domain and frequency-domain parameters, as described below.

Time-domain parameters include the standard deviation of all normal-to-normal intervals (SDNN), the square root of the mean of the sum of the square of differences between adjacent normal to the normal interval (RMSSD), and the proportion of adjacent sinus RR intervals differing by more than 50 ms (pNN50). SDNN is an overall indicator of HRV and reflects both sympathetic and parasympathetic activities, whereas RMSSD and pNN50 are related to parasympathetic activity. Frequency-domain parameters include the total power (TP), very low frequency (VLF), low frequency (LF), high frequency (HF), and the ratio of LF to HF (LF/HF). TP indicates the overall HRV. VLF is proposed to be sympathetic activity; LF indicates sympathetic activity; HF reflects parasympathetic activity; LF/HF measures the balance between sympathetic and parasympathetic activity. A higher LF/HF indicates greater sympathetic activity, while a lower ratio suggests increased parasympathetic activity [48,49].

Participants were instructed to refrain from stimulants (alcohol, caffeine, etc.) and exercise for 12 hours and food for 2 hours before the examination. During the entire measurement, participants had to remain silent and retain an empty bladder while breathing normally.

#### Cognitive function assessment.

Cognitive function was assessed using the Mini-Mental State Examination (MMSE), a widely used and well-validated screening tool. The MMSE consists of a series of questions that assess orientation, attention, memory, language, and visuospatial abilities. The total score ranges from 0 to 30, with a score of 24 and above indicating normal cognitive function [50]. The MMSE has been validated for use in various populations and languages, and it has been shown to have good sensitivity and specificity in detecting cognitive decline in clinical and community settings [51,52].

### 2.4 Statistical analysis

Data were presented as mean ± standard deviation (SD). The distributions of continuous variables were evaluated by the Kolmogorov-Smirnoff test. The variables of body temperature, systolic blood pressure, oxygen saturation, BMI, neck circumference, MMSE score, PSQI score, HADS scores, all WHOQOL-BREF scores, and all HRV indices were not normally distributed. In each analysis, missing data were excluded on a case-by-case basis. Differences between the groups in categorical variables were tested by the *χ*^2^ test. Differences in continuous variables were assessed by the Student’s *t*-test, Mann-Whitney *U*-test, or one-way ANOVA, as appropriate. To avoid the accumulation of type 1 errors across the variables, *p* values were adjusted using an adaptive linear step-up method that controls for a false discovery rate [53]. Effect sizes for significant differences in the values were calculated using Cramer’s *V*, Cohen’s *d*, or rank-biserial correlation, as appropriate. Correlation analyses between continuous variables were performed using Spearman’s bivariate test. Binary logistic regression was used to determine predictive factors associated with obesity (the dichotomized BMI score at 30 or higher). To assess the predictive accuracy of our model, we evaluated the area under the receiver operating characteristic curve. Linear regression was performed to determine if obesity and other psychosocial factors were associated with autonomic and cognitive function. The LF/HF and MMSE score were taken as dependent variables while independent variables were body measures, including BMI, and all potential confounders (sociodemographic factors, lifestyle determinants, psychological conditions, and quality of life). Multicollinearity was examined using variance inflation factor (VIF) and tolerance (1 < VIF < 2.5; tolerance<10). Homoscedasticity was assessed using scatter plots of residuals by predicted values. No outliers were detected (Cook’s distance, < 1; standard residuals <±3.3). The independence assumption was tested using the Durbin–Watson coefficient (satisfied if 1.5 < Durbin–Watson<2.5). A generalized linear model (GLM) was used to analyze if the relationship between obesity and cognitive decline is mediated by HRV indices. All statistical tests were two-tailed with a statistical significance set at *p* < 0.05. Data were analyzed using SPSS v26.0 and JAMOVI v2.2.5.

### 2.5 Ethical consideration

Written informed consent was obtained from all participants. All procedures performed in this study were done so in accordance with the ethical standards of the institutional and/or national research committee and the 1964 Helsinki Declaration and its later amendments. The design and methods were reviewed and approved by the Research Ethics Committee at the Mongolian National University of Medical Sciences, Ulaanbaatar, Mongolia (Approval number: 2020/03–05/#17).

### 2.6 Inclusivity in global research

Additional information regarding the ethical, cultural, and scientific considerations specific to inclusivity in global research is included in the Supporting Information (S1 Checklist).

## 3 Results

### 3.1 The prevalence of obesity and sociodemographic characteristics

This study included 382 participants (116 males and 266 females) aged 18–65 years with a mean ± SD of 41.0 ± 10.7 years. The details of the sociodemographic characteristics are described in Table 1: 69.6% were women; 73.6% were married; 29.6% held a bachelor’s degree or above; 59.2% were employed.

**Table 1:** Sociodemographic characteristics of the participants by sex.

Characteristics, n (%)		Male	Female	Total
All participants		116 (30.4)	266 (69.6)	382 (100)
Age group, 382	18-29	30 (25.9)	26 (9.8)	56 (14.7)
	30-44	41 (35.3)	137 (51.5)	178 (46.6)
	45-65	45 (38.8)	103 (38.7)	148 (38.7)
Marital status, 382	Never-married	13 (11.2)	33 (12.4)	46 (12.0)
	Others*	23 (19.8)	32 (12.0)	55 (14.4)
	Married	80 (69.0)	201 (75.6)	281 (73.6)
Education, 382	Secondary education and below	53 (45.7)	110 (41.4)	163 (42.7)
	Associate’s degree	25 (21.6)	81 (30.5)	106 (27.7)
	Bachelor’s degree	34 (29.3)	66 (24.8)	100 (26.2)
	Master’s degree and above	4 (3.4)	9 (3.4)	13 (3.4)
Employment, 382	Unemployed	13 (11.2)	52 (19.5)	64 (16.7)
	Student	14 (12.1)	13 (4.9)	27 (7.1)
	Pensioner	19 (16.4)	45 (16.9)	65 (17.0)
	Employed	70 (60.3)	156 (58.6)	226 (59.2)
Income, 382	<₮500,000	60 (51.7)	166 (62.4)	226 (59.2)
	₮500,001 - ₮1,500,000	51 (44.0)	98 (36.8)	149 (39.0)
	> ₮1,500,000	5 (4.3)	2 (0.8)	7 (1.8)
Workplace safety, 382	Safe	77 (66.4)	185 (69.5)	262 (68.6)
	Conditionally safe	32 (27.6)	76 (28.6)	108 (28.2)
	Potentially unsafe	2 (1.7)	4 (1.5)	6 (1.6)
	Hazardous	5 (4.3)	1 (0.4)	6 (1.6)
Living condition, 382	Ger (traditional pelt tent)	27 (23.3)	95 (35.7)	122 (31.9)
	House	42 (36.2)	83 (31.2)	125 (32.7)
	Dormitory	7 (6.0)	7 (2.6)	14 (3.7)
	Apartment	40 (34.5)	81 (30.5)	121 (31.7)
Residency location, 382	Urban	47 (40.5)	124 (46.6)	171 (44.8)
	Rural	69 (59.5)	142 (53.4)	211 (55.2)
Alcohol use, 346	Yes	36 (37.5)	91 (36.4)	127 (36.7)
	No	60 (62.5)	159 (63.6)	219 (63.3)
Tobacco use, 351	Yes	40 (42.6)	29 (11.3)	69 (19.7)
	No	50 (53.2)	225 (87.5)	275 (78.3)
	Had smoked before	4 (4.3)	3 (1.2)	7 (2.0)
Physical activity, 346	Never	12 (12.8)	44 (17.5)	56 (16.2)
	Rarely	7 (7.4)	31 (12.3)	38 (11.0)
	Sometimes	21 (22.3)	59 (23.4)	90 (23.1)
	Often	31 (33.0)	59 (23.4)	90 (26.0)
	Always	23 (24.5)	59 (23.4)	82 (23.7)
Vegetable consumption, 351	Never	1 (1.1)	7 (2.7)	8 (2.3)
	Rarely	7 (7.4)	28 (10.9)	35 (10.0)
	Sometimes	28 (29.8)	64 (24.9)	92 (26.2)
	Often	39 (41.5)	118 (45.9)	157 (44.7)
	Always	19 (20.3)	40 (15.6)	59 (16.8)
Fruit consumption, 355	Never	3 (3.1)	6 (2.3)	9 (2.5)
	Rarely	28 (29.2)	61 (23.6)	89 (25.1)
	Sometimes	57 (59.4)	160 (61.8)	217 (61.1)
	Often	8 (8.3)	23 (8.9)	31 (8.7)
	Always	0 (4)	9 (3.5)	9 (2.5)
Allergy food, 382	Yes	4 (3.4)	11 (4.1)	15 (3.9)
	No	112 (96.6)	255 (95.9)	367 (96.1)
Continuous variables, mean ± SD				
Age	Years, 382	39.5 ± 13.4	41.6 ± 9.3	41.0 ± 10.7
Vital function indices	Body temperature, 378	36.5 ± 0.5	36.5 ± 0.5	36.5 ± 0.5
	Heart rate, 376	80.5 ± 12.0	78.4 ± 11.0	79.1 ± 11.4
	SBP, 373	121.9 ± 18.8	126.4 ± 19.6	125.0 ± 19.5
	DBP, 373	76.8 ± 14.1	80.8 ± 12.0	79.6 ± 12.8
	AOS, 378	94.8 ± 2.2	94.7 ± 2.5	94.8 ± 2.4
Body measures	Neck circumference (cm), 361	37.7 ± 3.9	34.0 ± 2.7	35.0 ± 3.5
	Waist circumference (cm), 360	90.7 ± 14.3	89.8 ± 13.4	90.0 ± 13.6
	Weight (kg), 382	74.0 ± 15.5	69.3 ± 15.2	70.7 ± 15.0
	Height (cm), 382	167.2 ± 8.3	156.6 ± 6.3	159.8 ± 8.5
	BMI (kg/m^2^), 382	26.4 ± 5.1	28.3 ± 5.6	27.7 ± 5.5

* Others included remarried, co-habiting, separated, divorced, and widowed. ₮: Mongolian tugrik (MNT₮), US $1 = MNT ₮2850. Abbreviations: AOS: Arterial Oxygen Saturation. BMI: Body Mass Index. DBP: Diastolic Blood Pressure. n: number. SBP: Systolic Blood Pressure. SD: standard deviation.

There was missing data on alcohol use (9.4%), tobacco use (8.1%), physical activity (9.4%), vegetable consumption (8.1%), fruit consumption (7%), body temperature and arterial oxygen saturation (1%), heart rate (1.6%), systolic blood pressure and diastolic blood pressure (2.4%), neck circumference (5.5%), and waist circumference (5.8%).

The prevalence of obesity (BMI ≥ 30) was 32.2% in the study participants (Table 2). Because the participants had more females and were older, we adjusted age-sex for further analyses by weighting with the population report from the 2020 Population and Housing By-Census of Mongolia [33]. The age and sex-adjusted prevalence of obesity in the study population was 28.1%.

**Table 2:** The prevalence of obesity and socio-demographic and lifestyle factors by obesity.

Characteristics		Obesity		UPV	FPV	Effect size
		Yes	No			
Number (%)		123 (32.2)	259 (67.8)			
Prevalence: unadjusted: 32.2%; age-adjusted: 30.1%; sex-adjusted: 27.3%; age-sex-adjusted: 28.1%						
Categorical variables (%)^*^						
Sex	Male	28 (22.8)	88 (34.0)	**0.026**	0.056	
	Female	95 (77.2)	171 (66.0)			
Age group	18-29	6 (4.9)	50 (19.3)	**< 0.001**	**< 0.001**	0.239
	30-44	52 (42.3)	126 (48.6)			
	45-65	65 (52.8)	83 (32.1)			
Marital status	Never-married	17 (13.8)	29 (11.2)	**< 0.001**	**0.003**	0.219
	Others^§^	4 (3.3)	51 (19.7)			
	Married	102 (82.9)	179 (69.1)			
Education	Secondary education and below	55 (44.7)	108 (41.7)	**0.014**	**0.033**	0.167
	Associate’s degree	43 (35.0)	63 (24.3)			
	Bachelor’s degree	20 (16.3)	80 (30.9)			
	Master’s degree and above	5 (4.1)	8 (3.1)			
Employment	Unemployed	26 (21.1)	39 (15.1)	**0.002**	**0.005**	0.199
	Student	3 (2.4)	24 (9.3)			
	Pensioner	30 (24.4)	34 (13.1)			
	Employed	64 (52.1)	162 (62.5)			
Income	<₮500,000	77 (62.6)	149 (57.5)	NS	NS	0.095
	₮500,001 - ₮1,500,000	42 (34.1)	107 (41.3)			
	> ₮1,500,000	4 (3.3)	3 (1.2)			
Workplace safety	Safe	75 (60.9)	187 (72.2)	**0.002**	**0.005**	0.199
	Conditionally safe	43 (35.0)	65 (25.1)			
	Potentially unsafe	0 (4)	6 (2.3)			
	Hazardous	5 (4.1)	1 (0.4)			
Living condition	Ger (traditional pelt tent)	44 (35.8)	78 (30.1)	NS	NS	0.093
	House	37 (30.1)	88 (34.0)			
	Dormitory	2 (1.6)	12 (4.6)			
	Apartment	40 (32.5)	81 (33.3)			
Residency location	Urban	57 (46.3)	114 (44.0)	NS	NS	0.021
	Rural	66 (53.7)	145 (56.0)			
Alcohol use	Yes	40 (35.7)	87 (37.2)	NS	NS	0.014
	No	72 (64.3)	147 (62.8)			
Tobacco use	Yes	17 (14.9)	52 (21.9)	NS	NS	0.085
	No	95 (83.3)	180 (75.9)			
	Had smoked before	2 (1.8)	5 (2.1)			
Physical activity	Never	27 (24.8)	29 (12.2)	**< 0.001**	**< 0.001**	0.243
	Rarely	19 (17.4)	19 (8.0)			
	Sometimes	25 (22.9)	55 (23.2)			
	Often	21 (19.3)	69 (29.1)			
	Always	17 (15.6)	65 (27.4)			
Vegetable consumption	Never	5 (4.5)	3 (1.3)	NS	NS	0.132
	Rarely	7 (6.3)	28 (11.7)			
	Sometimes	31 (27.9)	61 (25.4)			
	Often	48 (43.2)	109 (45.4)			
	Always	20 (18.0)	39 (16.3)			
Fruit consumption	Never	1 (0.9)	8 (3.3)	NS	NS	0.120
	Rarely	31 (27.7)	58 (23.9)			
	Sometimes	67 (59.8)	150 (61.7)			
	Often	8 (7.1)	23 (9.5)			
	Always	5 (4.5)	4 (1.6)			
Food allergy	Yes	2 (1.6)	13 (5.0)	NS	NS	0.131
	No	121 (98.4)	246 (95.0)			
Continuous variables, mean ± SD ^#^						
Age	Years	44.9 ± 9.2	39.1 ± 10.9	**< 0.001**	**< 0.001**	0.561
Vital function indices	Body temperature	36.5 ± 0.5	36.5 ± 0.5	NS	NS	0.021
	Heart rate	78.4 ± 11.1	79.3 ± 11.5	NS	NS	0.080
	SBP	125.6 ± 18.0	124.8 ± 20.1	NS	NS	0.041
	DBP	81.1 ± 14.1	78.9 ± 12.1	NS	NS	0.172
	AOS	94.7 ± 2.5	94.8 ± 2.4	NS	NS	0.026
Body measures	Neck circumference	37.3 ± 3.5	33.9 ± 2.9	**< 0.001**	**< 0.001**	1.079
	Waist circumference	103.5 ± 9.8	83.7 ± 10.2	**< 0.001**	**< 0.001**	1.969
	Weight	85.4 ± 12.5	63.8 ± 10.2	**< 0.001**	**< 0.001**	1.965
	Height	158.5 ± 7.6	160.4 ± 8.9	**0.031**	NS	0.223
	BMI	33.9 ± 4.0	24.7 ± 3.2	**< 0.001**	**< 0.001**	2.645

* *p* values were analyzed with the *χ*^*2*^ test, and ^#^ Student’s *t*-test. ^§^ Others included remarried, co-habiting, separated, divorced, and widowed. ^₮^: Mongolian tugrik (MNT₮), US $1 = MNT ₮2850. Abbreviations: AOS: Arterial Oxygen Saturation. BMI: Body Mass Index. DBP: Diastolic Blood Pressure. n: number. NS: not significant. FPV: FDR-adjusted *p* values for multiple comparisons. SBP: Systolic Blood Pressure. SD: standard deviation. UPV: unadjusted *p* values.

The prevalence of central obesity was 64.2% in the study participants (22 missing values). After adjusting for age and sex, the prevalence of central obesity in the study population was 54.7%. There was a significant difference in central obesity between sexes (*χ*^*2*^(1) = 24.1, *p* < 0.001).

### 3.2 Lifestyle characteristics

As shown in Table 2, there were differences in the distribution of age group, marital status, education, employment, workplace safety, and physical activity between the participants with obesity and without obesity. Individuals with obesity were more likely to be older, married, have lower education and less physical activity. All body measurements differed between the groups, with the exception of the height.

### 3.3 Health-related quality of life

Health-related quality of life was measured with the WHOQOL-BREF. The mean scores of physical, psychological, social, and environmental quality of life were not different between the groups with or without obesity (Table 3).

**Table 3:** Health-related quality of life, mental health and autonomic dysfunction, and cognitive decline in individuals with and without obesity.

Characteristics		Obesity		UPV	FPV	Effect size
		Yes	No			
Health-related quality of life: (WHOQOL-BREF)	Physical domain	60.1 ± 14.5	60.9 ± 14.1	NS	NS	0.024
	Psychological domain	71.4 ± 14.1	72.2 ± 13.3	NS	NS	0.029
	Social domain	67.9 ± 16.3	68.9 ± 16.4	NS	NS	0.007
	Environmental domain	65.5 ± 15.9	65.1 ± 13.8	NS	NS	0.042
Psychological symptoms: (HADS)	Anxiety score	6.7 ± 3.4	6.8 ± 3.4	NS	NS	0.019
	Depression score	6.2 ± 3.0	5.9 ± 2.9	NS	NS	0.063
Sleep dysfunction: (PSQI) (BQ)	PSQI total score	9.1 ± 2.4	9.1 ± 2.1	NS	NS	0.014
	BQ - No risk	1 (0.8)	47 (18.1)	**< 0.001** ^*^	**<0.001**	0.363
	BQ - Low risk BQ - High risk	22 (17.9)	97 (37.5)			
		100 (81.3)	115 (44.4)			
Autonomic dysfunction: (HRV indices)	VLF	335.6 ± 534.0	395.8 ± 411.2	**< 0.001**	**< 0.001**	0.232
	LF	189.1 ± 367.5	250.6 ± 278.0	**< 0.001**	**< 0.001**	0.239
	HF	178.3 ± 423.1	180.8 ± 270.5	**0.006**	**0.007**	0.174
	LF/HF	2.3 ± 2.3	2.3 ± 2.1	NS	NS	0.047
	TP	704.0 ± 1219.4	828.2 ± 756.2	**< 0.001**	**< 0.001**	0.238
	SDNN	30.7 ± 14.5	36.2 ± 14.2	**< 0.001**	**< 0.001**	0.248
	RMSSD	22.9 ± 14.6	26.3 ± 13.5	**0.001**	**0.002**	0.206
	NN50	22.8 ± 39.8	26.5 ± 34.9	**0.001**	**0.002**	0.200
	pNN50	7.1 ± 12.7	8.6 ± 12.0	**0.002**	**0.003**	0.196
Cognitive decline: (MMSE)	MMSE total score	26.0 ± 3.9	26.9 ± 3.3	**0.036**	**0.038**	0.131

*p* values were analyzed with the Mann-Whitney *U*-test, with exceptions of

* : *χ*^*2*^ test. Abbreviations: BQ: Berlin questionnaire. FPV: FDR-adjusted *p* values for multiple comparisons. HADS: Hospital Anxiety and Depression Scale. HF: High-frequency. HRV: Heart Rate Variability. LF: Low-frequency. LF/HF: Ratio of low-frequency to high frequency. MMSE: Mini Mental State Examination. n: number. NN50: Number of consecutive NN intervals that varied by more than 50 ms. NS: not significant. pNN50: NN50 divided by the total number of NN intervals. RMSSD: Root mean square of the sum of the squares of differences between adjacent NN intervals. PSQI: Pittsburg Sleep Quality Index. SD: standard deviation. SDNN: Standard deviation of the normalized R-to-R (NN) intervals. TP: Total power. UPV: unadjusted *p* values. VLF: Very low-frequency. WHOQOL-BREF: World Health Organization Quality of Life Questionnaire Brief version.

### 3.4 Psychological conditions

Psychological conditions were assessed using self-report questionnaires for psychological symptoms and sleep dysfunction. Psychological symptoms, including anxiety and depression, were assessed with HADS. The mean anxiety and depression scores for both groups were within the normal range (scores < 8) and exhibited no significant differences by obesity (Table 3).

### 3.5 Sleep dysfunction

Sleep function was evaluated with PSQI and BQ. PSQI results suggest that sleep quality did not differ between the groups. In contrast, BQ results demonstrated that individuals with obesity were at increased risk of sleep apnea compared to individuals without obesity (Table 3).

### 3.6 Autonomic dysfunction

Autonomic function was measured using HRV parameters, which were compared between participants with obesity and without individuals (Table 3). In the time-domain parameters, all indices, including SDNN, RMSDD, and pNN50 were lower in individuals with obesity compared with individuals without obesity. In the frequency-domain parameters, individuals with obesity showed lower TP, VLF, LF, and HF values than individuals without obesity; only LF/HF did not differ between groups. These results indicate that individuals with obesity have decreased autonomic nervous system activity.

### 3.7 Cognitive decline

Cognitive function was assessed using MMSE. As shown in Table 3, the mean MMSE total scores of both groups were higher than 24. However, individuals with obesity showed reduced total scores compared to individuals without obesity.

### 3.8 Correlations between obesity-related measurements and autonomic and cognitive functions

As shown in Table 4, BMI and waist circumference were inversely correlated with all HRV indices, except LF/HF, in the study population.

**Table 4:** Spearman correlation coefficients between the HRV components and selected factors.

Variables	VLF	LF	HF	LF/HF	TP	SDNN	RMSSD	NN50	pNN50
All participants, n = 382									
BMI	-0.192^***^	-0.215^***^	-0.188^***^	0.008	-0.223^***^	-0.220^***^	-0.193^***^	-0.192^***^	-0.184^***^
Waist circumference	-0.166^**^	-0.212^***^	-0.214^***^	0.042	-0.220^***^	-0.225^***^	-0.235^***^	-0.214^***^	-0.210^***^
Neck circumference	-0.058	-0.060	-0.205^***^	0.234^***^	-0.105^*^	-0.109^*^	-0.207^***^	-0.185^**^	-0.177^**^
Weight	-0.126^*^	-0.124^*^	-0.176^**^	0.086	-0.155^**^	-0.147^**^	-0.168^**^	-0.154^**^	-0.147^**^
Height	0.117^*^	0.137^**^	0.001	0.134^**^	0.104^*^	0.102*	-0.005	0.016	0.019
Age	-0.224^***^	-0.349^***^	-0.254^***^	-0.054	-0.297^***^	-0.320^***^	-0.293^***^	-0.305^***^	-0.290^***^
Heart rate	0.106^*^	0.062	0.061	-0.025	0.099	0.096	0.061	0.047	0.049
SBP	-0.060	-0.085	-0.029	-0.052	-0.068	-0.059	-0.004	0.026	0.018
WHOQOL-BREF Physical domain	0.122^*^	0.092	0.042	0.059	0.096	0.106^*^	0.055	0.049	0.051
WHOQOL-BREF Psychological domain	0.042	0.017	-0.054	0.102^*^	0.015	0.025	-0.029	-0.020	-0.014
HADS Depression score	-0.079	-0.106^*^	-0.047	-0.053	-0.087	-0.086	-0.046	-0.051	-0.061
Participants with obesity, n = 123									
Waist circumference	-0.070	-0.161	-0.195^*^	0.096	-0.135	-0.139	-0.192^*^	-0.094	-0.089
Neck circumference	-0.153	-0.163	-0.285^**^	0.254^**^	-0.207^*^	-0.227^*^	-0.355^***^	-0.253^**^	-0.252^**^
Age	-0.121	-0.217^*^	-0.202^*^	0.033	-0.176	-0.215^*^	-0.238^**^	-0.235^*^	-0.231^*^

* *p* < 0.05,

** p < 0.01,

*** p < 0.001. *p* values were analyzed using the Spearman’s correlation. Abbreviations: BMI: Body Mass Index. HADS: Hospital Anxiety and Depression Scale. HF: High-frequency. LF: Low-frequency. LF/HF: Ratio of low-frequency to high frequency. NN50: Number of consecutive NN intervals that varied by more than 50 ms. RMSSD: Root mean square of the sum of the squares of differences between adjacent NN intervals. pNN50: NN50 divided by the total number of NN intervals. SBP: Systolic Blood Pressure. SD: standard deviation. SDDN: Standard deviation of the normalized R-to-R (NN) intervals. TP: Total power. VLF: Very low-frequency.WHOQOL-BREF: World Health Organization Quality of Life Questionnaire Brief version.

Neck circumference was inversely correlated with SDNN, RMSSD, pNN50, HF, and TP, whereas it positively correlated with LF/HF. Subgroup analysis showed that waist circumference inversely correlated with RMSSD and HF in individuals with obesity. Neck circumference inversely correlated with SDNN, RMSSD, pNN50, HF, and TP, but positively correlated with LF/HF. Additionally, age is inversely correlated with almost all HRV indices.

### 3.9 Risk factors that are associated with obesity

A logistic regression was performed to determine the associations of sociodemographic and lifestyle factors, health related quality of life and psychological characteristics, sleep dysfunction, body measurements, autonomic, and cognitive function on the likelihood that participants have obesity (Table 5).

**Table 5:** Binary logistic regression on selected risk factors associated with obesity.

Characteristics	Estimate	SE	z	*p* value	OR	VIF	Tolerance
Model fit summary: *χ*^*2*^(34) = 217; *p* < 0.001; Nagelkerke *R*^*2*^ = 0.831							
Intercept	-75.525	31.839	-2.372	0.018			
Sociodemographic characteristics							
Sex (reference: male)	6.228	1.857	3.354	< 0.001	5.67	8.78	0.117
Education	-1.483	0.595	-2.489	0.013	0.23	2.99	0.334
Living condition	0.904	0.397	2.276	0.023	2.47	3.01	0.333
Alcohol use	2.540	0.946	2.686	0.007	12.68	2.60	0.385
Body measures							
Neck circumference	0.512	0.204	2.516	0.012	1.68	4.72	0.212
Waist circumference	0.397	0.087	4.582	< 0.001	1.49	5.03	0.199
Sleep dysfunction							
Sleep apnea (BQ)	2.556	1.08	2.366	0.018	12.87	2.66	0.376
Autonomic function (HRV indices)							
TP	-0.001	9.25	-2.138	0.033	1.0	6.35	0.158
LF/HF	0.543	0.228	2.382	0.017	1.72	2.82	0.355
HF	0.009	0.003	2.914	0.004	1.01	8.58	0.117

Abbreviations: BQ: Berlin questionnaire. HF: High-frequency. HRV: Heart rate variability. LF: Low-frequency. LF/HF: Ratio of low-frequency to high frequency. OR: Odds ratio. SE: Standard error. TP: Total power. VIF: Variance inflation factor. VLF: Very low-frequency. Z: Regression coefficient.

**Fig 2:**
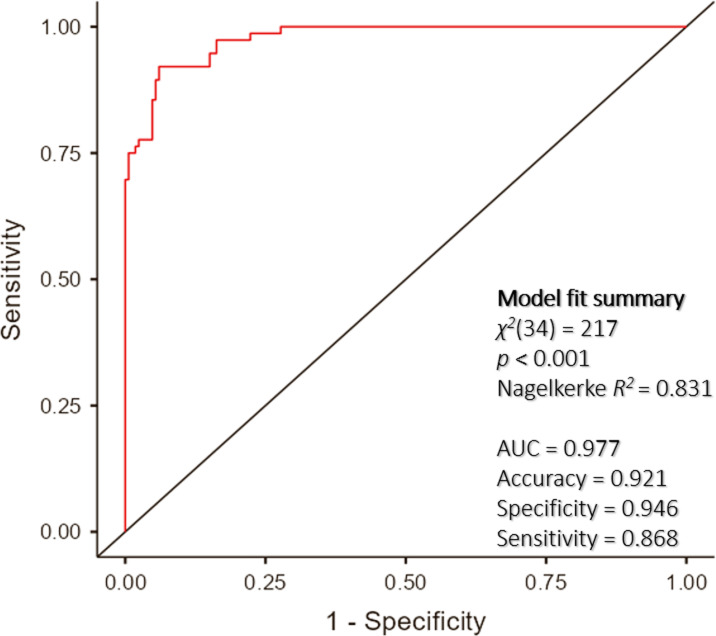
ROC curve of the predictive factors for obesity. The area under the receiver operating characteristic curve was 0.977, reflecting high predictive accuracy of 0.921, with a specificity of 0.946 and a sensitivity of 0.868.

The logistic regression model demonstrated statistical significance, with *χ*^2^(34) = 217 and p < 0.001. This model accounted for 83.1% of the variance in obesity, as indicated by the Nagelkerke *R*^*2*^ value, and accurately classified 92.1% of cases (Fig 2).

As shown in Table 5, females (odds ratio [OR]: 5.67), those with a secondary or lower education level (OR: 0.2), those who lived in an apartment (OR: 2.5), and those who used alcohol (OR: 12.7) were more likely to exhibit obesity compared with those who were male, had a higher education level, lived in gers or houses, and did not use alcohol. Increasing neck circumference (OR: 1.7), waist circumference (OR: 1.5), and sleep apnea (OR: 12.9) were associated with an increased likelihood of exhibiting obesity. In HRV parameters, increasing LF/HF (1.7) and HF (1.0) but decreasing TP (OR: 1.0) was associated with a rise in the likelihood of exhibiting obesity. This suggests that obesity is associated with autonomic imbalance, increased parasympathetic activity, and overall decreased autonomic function.

### 3.10 Effects of obesity on autonomic and cognitive functions

To investigate how autonomic dysfunction and cognitive decline were independently predicted by the risk factors in the sample, linear regression analysis was performed on LF/HF and MMSE scores, separately (S2 Table). The results demonstrate that LF/HF was associated with SBP, physical activity, and neck circumference. The MMSE score was predicted by age, education, height, sleep apnea, LF/HF, and SDNN. No multicollinearity was detected between the tested variables; the independence assumption was satisfied; the distribution of the residuals satisfied the normality assumptions. The variance of the model was constant, and homoscedasticity was not violated.

Furthermore, within the participants with obesity subgroup, lower heart rate variability and lower cognitive test scores were associated with worse physical quality of life, lower physical activity levels, and higher BMI. These findings suggest that cognitive decline in people with obesity have overall decreased autonomic function.

Additionally, the GLM mediation analysis indicated that there was a significant direct and indirect relationship between obesity and cognitive decline. The indirect relationship was mediated by decreased SDNN and increased RMSSD (S3 Fig).

Overall, these results suggest a potential mediation effect of autonomic dysfunction on the association between obesity and cognitive decline.

## 4 Discussion

In this study, we investigated the prevalence of obesity and its association with autonomic dysfunction, cognitive decline, sleep dysfunction, psychological symptoms, and health-related quality of life among 382 Mongolian adults aged 18–65 years. The results demonstrated that 32.2% of the participants had obesity, and the age and sex-adjusted prevalence of obesity in the general population was estimated at 28.1%, indicating a slightly higher prevalence of obesity in our study population than the general population worldwide [54]. Given that the study was conducted in 2020, it is possible that the prevalence was affected by the ongoing pandemic, as obesity has been identified as a substantial risk factor for complications and mortality [55,56]. Furthermore, the prevalence of central obesity in our study population was relatively higher than the global average of 41.5% [5].

The results also revealed that an association between obesity and autonomic dysfunction, as evidenced by reduced HRV parameters (SDNN, RMSDD, pNN50, TP, VLF, LF, and HF). These findings align with previous studies that have suggested a link between obesity and autonomic dysfunction [57,58], possibly due to increased adiposity leading to inflammation and insulin resistance, which can negatively affect autonomic function [59].

Regarding cognitive decline, our results showed a lower mean MMSE score in participants with obesity compared to participants without obesity. However, both groups had an average score above 24 in MMSE, which is considered normal. While this may suggest a minor impact of obesity on cognitive function, further research is needed to establish a more definitive relationship [60].

With regard to sleep dysfunction, no significant difference in sleep quality was observed between participants with obesity and without obesity based on the PSQI scores. This differs from the findings of our previous hospital-based study [61]. However, the BQ results indicated that individuals with obesity were at an increased risk for sleep apnea. This finding is supported by existing literature [62]. Sleep disturbances have been demonstrated to impair cognitive function [63]. Furthermore, previous studies have indicated potential associations between sleep duration and cognitive function in both younger and older populations, which may be mediated by testosterone levels [64–66].

Psychological symptoms, including anxiety and depression, did not differ between the two groups, as assessed by the HADS scores. In this study, we used the HADS to evaluate psychological symptoms of obesity. However, the HADS does not assess physical symptoms such as pain, fatigue, or gastrointestinal concerns. Obesity can manifest as both psychological and physical symptoms. Therefore, future studies should use more sensitive tools that capture both physical and mental symptoms, such as the Brain Overwork Scale [67].

Furthermore, health-related quality of life, measured using the WHOQOL-BREF, showed no significant differences in physical, psychological, social, or environmental domains between individuals with obesity and without obesity. This is in line with some previous research findings [68], although other studies have reported lower quality of life in individuals with obesity [10,69]

Our analysis revealed that BMI and waist circumference were inversely correlated with HRV indices, except for LF/HF, suggesting that increased adiposity is associated with reduced autonomic function. Moreover, neck circumference was found to be inversely correlated with several HRV indices, while positively correlated with LF/HF. These correlations could imply that central obesity plays a role in the dysregulation of autonomic function [13,16]. Additionally, age was inversely correlated with almost all HRV indices, indicating the potential influence of aging on autonomic function [70].

The logistic regression identified several risk factors associated with an increased risk of obesity, including female sex, living in an apartment, alcohol use, increased neck and waist circumferences, sleep apnea, and altered autonomic nervous system function. Of note, the increased LF/HF observed in the study suggests a possible association between the overactivity of the sympathetic nervous system and an increased risk of obesity. This overactivity may result in increased stress and inflammation, which can contribute to weight gain and obesity development. LF/HF is an important indicator of the balance between sympathetic (stress response) and parasympathetic (relaxation response) activity, with higher values indicating a shift towards sympathetic dominance. Furthermore, we noted a reduction in total power (TP) among individuals with obesity. As TP represents overall autonomic nervous system activity through frequency-domain analysis, this reduction may indicate dysregulation in both the sympathetic and parasympathetic activities. These findings suggest that autonomic dysfunction and central obesity are associated with an increased risk of obesity [71].

The linear regression identified key risk factors for autonomic dysfunction and cognitive decline, with evidence suggesting a partial mediation of the obesity-cognition link by autonomic dysfunction. We also found that individuals with obesity who had lower SDNN scores were at increased risk of cognitive decline. SDNN is a measure of the variability of the heart rate, and it is thought to be a marker of overall autonomic activity. The results of the linear regression analysis are consistent with the findings of the mediation analysis, which indicated a significant association between obesity and cognitive decline, mediated by autonomic dysfunction. The results demonstrate that interventions that improve autonomic dysfunction may be beneficial for cognitive health, as autonomic dysfunction may be an early marker of cognitive decline in people with obesity. However, the associations between obesity, autonomic dysfunction, and cognitive decline are complex and multifaceted. It has been suggested that autonomic dysfunction may contribute to the development of obesity, and conversely, that obesity can also lead to autonomic dysfunction [13,18,72]. It is important to note that obesity is often accompanied by sarcopenia [73], although this was not measured in the present study. Muscle loss, particularly in the context of sarcopenic obesity, can further exacerbate metabolic dysfunction, reducing insulin sensitivity and energy expenditure, thereby contributing to the development of obesity [74]. Autonomic dysfunction may influence hormonal pathways, such as cortisol and testosterone regulation, which are important for maintaining muscle mass [75]. The underlying mechanisms linking obesity, autonomic dysfunction, and sarcopenia are likely complex and involve multiple factors, including inflammation, oxidative stress, and hormonal dysregulation [76]. Further studies are needed to explore the underlying mechanisms that may contribute to this relationship and to identify potential interventions to mitigate the impact of obesity on the autonomic and cognitive function.

Several limitations of this study should be noted. First, the cross-sectional design of the study limits our ability to establish causality between obesity, autonomic dysfunction, and cognitive decline. Longitudinal studies would be needed to better understand the temporal relationships between these factors. Second, the sample size may not be large enough to detect subtle differences between participants with obesity and without obesity in specific measures. Additionally, the study population might not adequately represent the general population in western countries, potentially impacting the generalizability of our findings. Third, self-reported measures, such as the questionnaires used to assess sleep dysfunction, psychological symptoms, and quality of life, may be subject to recall bias and social desirability bias. Fourth, the study did not control for the presence of comorbidities or medications that could influence HRV parameters and cognitive function. Furthermore, the use of single measures, such as MMSE for cognitive function, may not capture the full extent of cognitive dysfunction associated with obesity [77]. Its brevity and ease of administration made it suitable for our study, particularly given the extensive battery of other assessments. However, to provide a more comprehensive evaluation of cognitive function in future research, it may be beneficial to incorporate additional neuropsychological tests that are more sensitive to mild cognitive impairment. Moreover, more detailed information on nutritional intake data, including macronutrient intake, would provide a more comprehensive understanding of the relationship between diet, obesity, and cognitive function.

In conclusion, our study highlights a high prevalence of obesity in the general population (28.1%) and identifies distinct characteristics associated with the condition. Furthermore, our findings suggest a potential indirect effect of obesity on cognitive function, mediated by autonomic dysfunction. Despite the limitations, these findings contribute to the growing body of literature on the consequences of obesity and emphasize the need for public health interventions to address the obesity epidemic. Further research is needed to elucidate the causal relationships and develop targeted interventions for high-risk groups (females, individuals with lower education) and promotion initiatives of healthy lifestyles (less alcohol, exercise, and sleep hygiene) to address both obesity and its associated health complications, including autonomic dysfunction.

## Supporting information

S1 File Inclusivity in global research. (DOCX)

S2 Table Linear regression analyses on the autonomic dysfunction and cognitive decline by risk factors. (DOCX)

S3 Fig GLM mediation model for the relationship between obesity and cognitive decline by mediation of the HRV indices. A generalized linear model (GLM) was performed to investigate if autonomic dysfunction mediates the effects of obesity on cognitive decline. HRV indices (time-domain and frequency-domain parameters were separately taken) were utilized as mediators, while the dichotomized BMI at 30 and the continuous MMSE score were taken as the independent and outcome variables, respectively. The mediation model indicates that the indirect pathways from obesity to cognitive decline through the HRV components showed a significant relationship (*p *= 0.038). Among the HRV components, SDNN and RMSSD-mediated pathways had significant correlations (*p *= 0.047 and *p *= 0.034, respectively).
(TIF)
