# Supplementary material for: Obesity and its associations with autonomic and cognitive functions in the general population
Source: PLoS One. 2025 May 8;20(5):e0322802. doi: 10.1371/journal.pone.0322802 (PMC12061429; doi:10.1371/journal.pone.0322802)
Supplement: S2 Table — (DOCX) [file pone.0322802.s002.docx]

**S2 Table.** Linear regression analyses on the autonomic dysfunction and cognitive impairment by risk factors.

| **Variables** | **B** | **SE** | **t** | ***p* value*** | **95% Confidence**  **Interval for Exp (B)** | | **Collinearity Statistics** | |
| --- | --- | --- | --- | --- | --- | --- | --- | --- |
|  |  |  |  |  | **Lower** | **Upper** | **Tolerance** | **Variance Inflation**  **Factor** |
| All participants, n = 382 | | | | | | | | |
| LF/HF (R^2^ = 0.266, Durbin-Watson = 0.110, F_31_ = 1.49, *p* = 0.031) | | | | | | | | |
| Constant | 10.08 | 9.79 | 1.03 | 0.305 | -9.23 | 29.39 |  |  |
| SBP | 0.02 | 0.01 | 2.14 | 0.034 | 0.001 | 0.04 | 0.56 | 1.78 |
| Physical activity | 0.90 | 0.40 | 2.27 | 0.024 | 0.12 | 1.68  4 | 0.89 | 1.13 |
| Neck circumference | 0.15 | 0.06 | 2.4 | 0.017 | 0.03 | 0.27 | 0.48 | 2.08 |
| MMSE (R^2^ = 0.259, Durbin-Watson = 0.067, F_17_ = 5.11, *p* < 0.001) | | | | | | | | |
| Constant | 13.47 |  | 2.29 | 0.023 | 1.90 | 25.03 |  |  |
| Age | -0.05 | -0.13 | -2.03 | 0.042 | -0.09 | -0.001 | 0.63 | 1.57 |
| Education | 0.70 | 0.16 | 2.78 | 0.006 | 0.20 | 1.20 | 0.81 | 1.22 |
| Height | 0.08 | 0.20 | 2.61 | 0.009 | 0.02 | 0.15 | 0.50 | 1.99 |
| BQ | -1.37 | -0.27 | -4.26 | < 0.001 | -2.01 | -0.74 | 0.73 | 1.36 |
| LF/HF | -0.29 | -0.14 | -2.57 | 0.011 | -0.51 | -0.06 | 0.88 | 1.13 |
| SDNN | -0.03 | -0.16 | -2.69 | 0.008 | -0.06 | -0.01 | 0.83 | 1.20 |
| Participants with obesity, n = 123 | | | | | | | | |
| LF/HF (R^2^ = 0.514, Durbin-Watson = 0.086, F_31_ = 1.78, *p* = 0.040) | | | | | | | | |
| WHOQOL-BREF  Physical domain | -0.06 | 0.03 | -2.18 | 0.035 | -0.12 | -0.005 | 0.57 | 1.76 |
| Physical activity | 2.84 | 0.87 | 3.28 | 0.002 | 1.10 | 4.59 | 0.82 | 1.21 |
| BMI | 0.27 | 0.11 | 2.44 | 0.019 | 0.05 | 0.50 | 0.49 | 2.03 |
| MMSE (R^2^ = 0.330, Durbin-Watson = 0.050, F_17_ = 2.35, *p* = 0.006) | | | | | | | | |
| Constant | 21.57 |  | 1.65 | 0.102 | -4.41 | 47.55 |  |  |
| Age | -0.11 | -0.25 | -2.27 | 0.026 | -0.20 | -0.01 | 0.67 | 1.49 |
| SDNN | -0.07 | -0.28 | -2.87 | 0.005 | -0.12 | -0.02 | 0.82 | 1.21 |

* *p* values were tested using the linear regression. Abbreviations: BMI: Body Mass Index. BQ: Berlin questionnaire. LF/HF: Ratio of low-frequency to high frequency. MMSE: Mini Mental State Examination. SBP: Systolic Blood Pressure. SE: Standard error. SDNN: Standard deviation of the normalized R-to-R (NN) intervals. WHOQOL-BREF: World Health Organization Quality of Life Questionnaire Brief version.
