# Supplementary figures and images for: Obesity and its associations with autonomic and cognitive functions in the general population
Source: PLoS One. 2025 May 8;20(5):e0322802. doi: 10.1371/journal.pone.0322802 (PMC12061429; doi:10.1371/journal.pone.0322802)

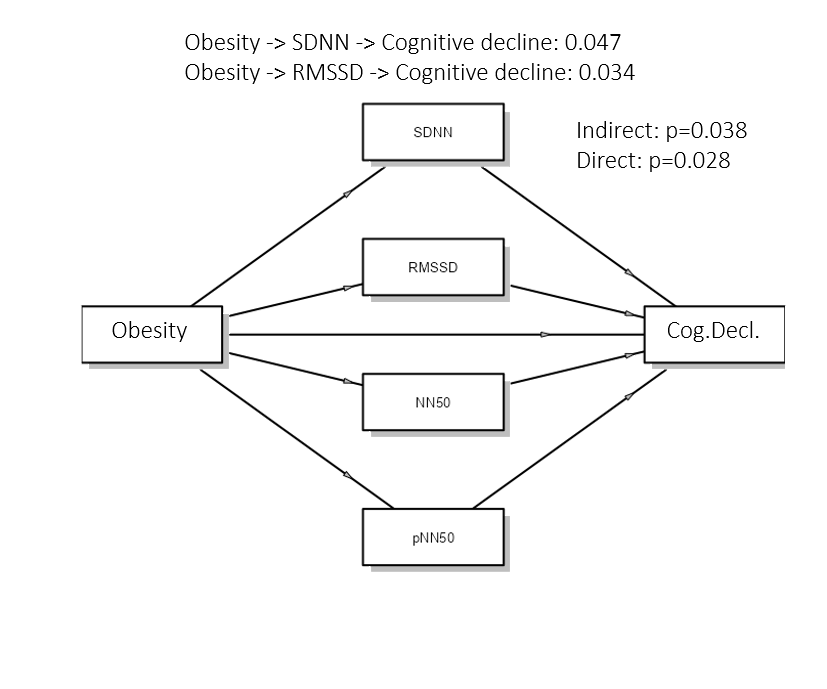

Supplement: S3 Fig — A generalized linear model (GLM) was performed to investigate if autonomic dysfunction mediates the effects of obesity on cognitive decline. HRV indices (time-domain and frequency-domain parameters were separately taken) were utilized as mediators, while the dichotomized BMI at 30 and the continuous MMSE score were taken as the independent and outcome variables, respectively. The mediation model indicates that the indirect pathways from obesity to cognitive decline through the HRV components showed a significant relationship (p = 0.038). Among the HRV components, SDNN and RMSSD-mediated pathways had significant correlations (p = 0.047 and p = 0.034, respectively). (TIF) [file pone.0322802.s003.tif]
